# Opinion: leading position of ultrasound in decision algorithm for small papillary thyroid carcinoma

**DOI:** 10.1186/s13244-022-01240-5

**Published:** 2022-06-07

**Authors:** Pierre Yves Marcy, Gilles Russ, Luca Saba, Julie Sanglier, Edouard Ghanassia, Haithem Sharara, Juliette Thariat, Jean Baptiste Morvan, Alain Bizeau

**Affiliations:** 1 Department of Radiodiagnostics and Interventional Imaging, Polyclinics ELSAN Group, PolyClinics Les Fleurs, Quartier Quiez, 83189 Ollioules, France; 2 Centre de Pathologie et d’Imagerie Paris 14ème, Unité Thyroïde et Tumeurs Endocrines. Hôpital La Pitie Salpetriere, 83 Bd de l’Hopital, Sorbonne Université, Paris, France; 3 Department of Radiology, Humanitas Mater Domini, Castellanza, VA Italy; 4 Department of Ultrasound, Antoine Lacassagne Cancer Research Institute, Sophia Antipolis University, 33 Avenue Valombrose, Nice Cedex, France; 5 American Hospital of Paris, 63 bd Victor Hugo, Neuilly sur Seine, France; 6 Department of Radiology, University Hospital, Place du Pr R. Debré, Nîmes Cedex 9, France; 7 Department of Radiation Oncology, Cancer Research Institute Francois Baclesse, 3 Avenue Général Harris, Caen, France; 8 Department of Head and Neck Surgery, University Military Hospital Sainte-Anne, 2, Boulevard Sainte Anne, BP 600, Toulon, France; 9 Department of Head and Neck Surgery, Sainte Musse Hospital, 54, Rue Henri Sainte Claire Deville, Toulon, France

**Keywords:** Watchful waiting, Radiofrequency ablation, Papillary thyroid cancer, Ultrasonography, Thyroid

## Key points

- Doppler US has a leading position in selecting patients with low-risk PTC.
- Multiparametric ultrasound assessment and appropriate settings are mandatory.
- Five PTC characteristics should be labelled and reported on standard scheme.
- Unfavourable factors, RLN palsy, suspicious US/FNA rule out active surveillance.
- Percutaneous RFA can be an option for patients eligible for active surveillance.

This Opinion article reports our view regarding the course of action to be taken when facing T1 papillary thyroid carcinoma, and the leading role of ultrasound in the decision algorithm.

This is an opinion letter and a reply letter to the article “Ultrasound in active surveillance for low-risk papillary thyroid cancer: imaging considerations in case selection and disease surveillance” published by Ghai S, et al. in Insights into Imaging [1].

Further to the paper entitled “Ultrasound in active surveillance for low-risk papillary thyroid cancer (PTC): imaging considerations in case selection and disease surveillance” recently published, we congratulate the authors Ghai et al. and would like to make further comments.

The baseline thyroid scans evaluated up to 4 nodules at study outset. PTC’s size in 3 dimensions, precise location of the nodule (along the course of recurrent laryngeal nerve abutting the trachea, upper, mid, lower third, isthmus, sub capsular, extrathyroidal extension) or evidence of nodal metastases were recorded. All patients had 6-month interval scan follow-up for the first 2 years and then yearly.

**Fig. 1:**
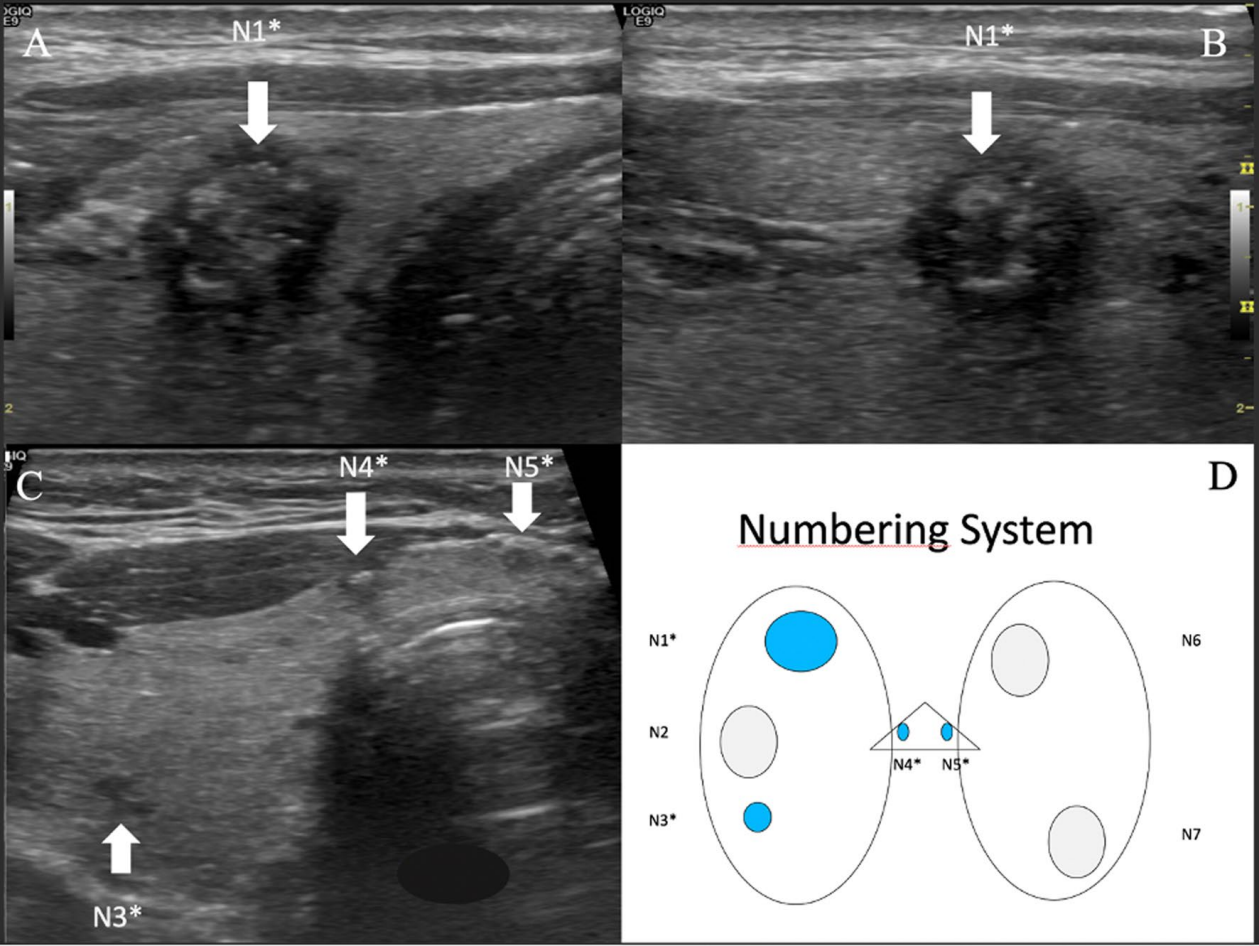
Thyroid nodule labelling and numbering at study outset (and follow- up). **A** Axial US scan of upper right lobe of thyroid gland. **B** Sagittal scan of right lobe. **C** Axial scan of right lobe and isthmus. **D** Diagram. Nodules are numbered from upper to lower lobe from right to left. In case of multifocal malignant nodule, various tumour foci are identified on US scan (**A**–**C**) as N1*, N3*, N4* and N5*, and reported on the diagram (**D**). Sum of each focus is calculated: 7 mm (upper right lobe) + 3 mm (lower right lobe) + 3 mm (isthmus) + 2 mm (isthmus) = 15 mm (pT1b) (**D**). Note that considering the only main tumour focus (N1) leads to pT1a diagnosis according to the TNM classification. Echogenic foci should be differentiated from comet tail artefacts and are considered as psammoma body microcalcifications and be reported

We agree with the authors for storing a key-image displaying the malignant thyroid nodule on PACS; we strongly advocate to report, on an additional standardised identification scheme (Fig. 1), the precise characteristics of the malignant nodule (pT1a vs pT1b, presence of multiple similar tumour foci, location, presence of hyperechoic foci suggestive of microcalcifications, extra thyroid extension ETE), the allocated Eu-TIRADS score, location of suspicious lymph node (s) according to the Robbins’s classification [2], and TNM classification. The distribution of microcalcifications in the thyroid gland should also be assessed. Widespread microcalcifications, especially when associated with suspicious cervical lymph nodes in a young patient < 20 years old are suspect of a diffuse sclerosing variant of PTC.

As reported by Ghai et al., we also note the presence of thyroiditis (reduction of thyroid gland echogenicity, reactive adenitis at level VI, positive serum anti-Thyroid peroxidase (ATPO) antibody level) when available, and label other non-suspicious nodules (Fig. 1). In lymphocytic thyroiditis and/or Grave’s disease settings, we agree with Ghai et al. regarding the difficulty of diagnosis and follow up of malignant hypoechoic nodules that may limit accurate reproducible measurements of thyroid nodules. Doppler US and microvascular imaging as well as elastography optimise delineation and accurate measurement in three dimensions of the target tumour [3]. To better characterise isoechoic suspicious nodules, one should decrease the dynamic range settings to increase the contrast ratio between the target nodule and the contiguous thyroid gland.

As auto-immune processes frequently render thyroid gland hypoechoic, the contrast with a hypoechoic nodule can be reduced or suppressed. Sometimes, the use of elastography can show a zone of focal increased stiffness corresponding to the nodule, therefore helping to detect and assert its existence. If strain elastography [4, 5] is applied, the use of a nodule-to-muscle ratio could be more helpful than a nodule-to-surrounding thyroid parenchyma, due to the modifications of the latter in chronic autoimmune thyroiditis.

It is worthy to note that the authors applied the rule of nodule measurement follow-up that was dedicated to T1a thyroid microcarcinomas (*T* < 10 mm) according to the Japan Association of Endocrine Surgery Task Force on management for papillary thyroid microcarcinoma [6], whereas the authors also included T1b tumours (10 mm < *T* < 20 mm) in their study [1]. Thus, any disease progression prompting surgical recommendation (> 3 mm in anyone dimension according to ACR TI-RADS nodule’ s orientation) represents a 15% cut-off long axis progression in T1b compared to 30% in T1a thyroid carcinomas [1, 6]. Moreover, the choice of using a single diameter and not a volume increase is debatable. Rozenbaum et al. [7] found a volume progression ≥ 50% in 35.0% (at any time during the follow-up) and 26.3% of patients (if considering first and last follow-up), whereas a diameter increase of ≥ 3 mm was seen in 3.8% of cases only. Thus, we advise the use of volume as a supplementary criterion of nodule increase, with a cut-off of 50%. To reduce interobserver variation, proceeding to multiple measurements of the same nodule usually allows to more accurate estimations. Noteworthy, we should keep in mind that thyroid US may overestimate by 2 mm the size of pT1a thyroid carcinomas [7], and by 19.5% the pT1b cystic like carcinomas size, when compared to the definitive gross histological size [8].

In case of macrocalcification, we use multiparametric ultrasonography assessment, shifting from lower to higher frequency ultrasonography (US) neck probe. Adjusting the bandwidth frequency of the US probe, focusing on suspicious area of interest (when not automatic) will help to better assess the nodule’s outlines and measure the target nodule.

**Fig. 2:**
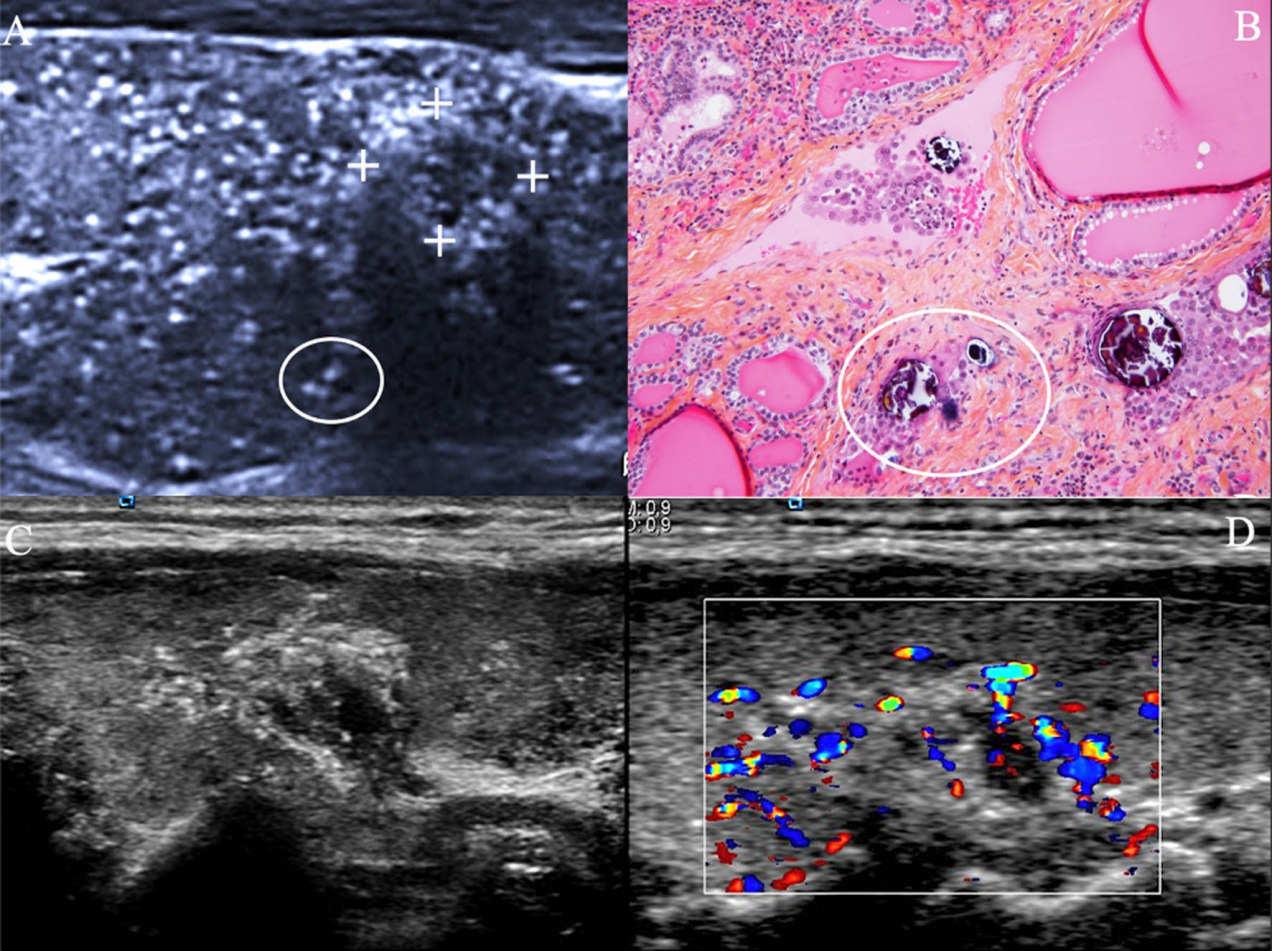
US scan of two diffuse Sclerosing Variants (DSV) of PTC. *Case 1* Longitudinal US scan of right lobe (**A**), histopathological specimen (**B**, 20 ×). DSV shows diffuse extensive psammoma bodies (O) of thyroid lobe (**A**, **B**), usually without forming a gross tumour nodule. In the present case, a dominant 9 mm Eu-TIRADS4 anterior nodule T1a is displayed (**A**, ++). *Case 2* Longitudinal US scan shows Eu- TIRADS5 posterior thyroid nodule (**C**); Doppler US discloses twinkling artefacts of psammoma microcalcifications on Doppler US (**D**). TNM staging: the radiologist takes into account the PTC nodule size, whereas the histopathologist takes into account the nodule size but also the long axis of microcalcifications, which correspond to intra lymphatic intralobar thyroid tumour spread

The distribution of microcalcifications in the thyroid gland should also be assessed. Widespread microcalcifications, especially when associated with suspicious cervical lymph nodes in a young patient are suspect of a diffuse sclerosing variant of PTC (DSV). Dispersed echogenic foci of PTC islands, the so-called psammoma bodies, metastasise into dilated lymphatics vessels (Fig. 2) and may represent a formal contraindication to active surveillance [9].

According to TNM eighth edition [10], gross extra-thyroidal extension invading strap muscles is considered as stage T3b, while invasion into soft tissues, larynx, trachea, oesophagus, recurrent laryngeal nerve (RLN) is staged T4a, whatever the tumour size.

**Fig. 3:**
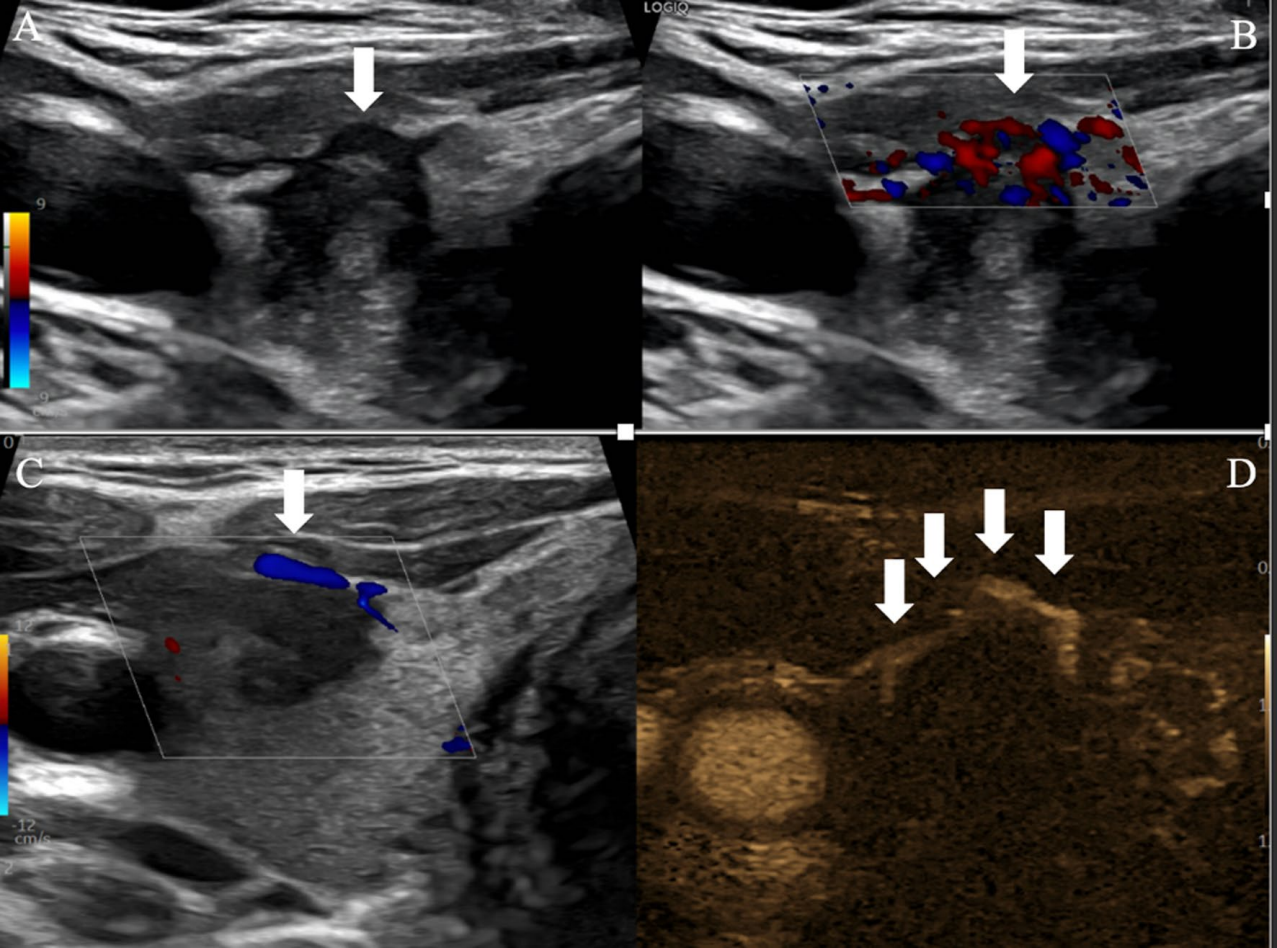
US assessment of PTC extra thyroid extension (ETE). Axial US scan, grey scale (**A**), colour Doppler (**B**, **C**), MicroVascular Imaging MVI (**D**) of right lobe PTC. Contour bulging is shown (arrow, **A**), neovasculature (arrow) is seen running on the capsula and increases sensitivity of ETE diagnosis (**B**). In case of anterior abutment without disruption (**C**, **D**), Doppler US discloses vessel (arrow) running along the, while MVI depicts more subtle capsular vessels (arrows)

Regarding extra-thyroidal extension, the authors excluded from the active surveillance the PTC presenting with ETE criteria. The latter include (a) abutment; (b) disruption, loss of perithyroid echogenic line at site of contact with the known cancerous nodule; (c) contour bulging, and (d) replacement of strap muscle, with indistinct margin with the strap muscle (for gross ETE). Interestingly, we noticed that Doppler US and novel microvascular imaging technique can be of significant value to predict ETE, namely when tiny vessels are shown running along or perpendicular to the thyroid nodule capsule [11]. Adjusting the field of view, the probe frequency bandwidth and the focus zone will increase thyroid capsular vessel flow imaging and subsequent prediction of ETE (Fig. 3). However, in posterior and deep located thyroid nodule, microvascular imaging (MVI) seems of limited value, and computed tomography (CT) should be considered as a further diagnosis step of ETE to the oesotracheal groove, as recommended by Ghai et al. [1].

Upper lobe location has been linked to a higher risk of lateral lymph node, skip metastasis [12], and lymph node recurrence, and should be considered at the initial description and be reported on the study outset scheme for further assessment of PTC (Fig. 1). According to Seok et al., PTC location at the isthmus comprises 4.6% of all PTCs and have more lymphatic invasion (22.1% vs. 13.4%), ETE (73.0% vs. 57.1%), and perithyroidal and prelaryngeal node metastasis (18.0% vs. 9.0%) compared to lobar PTCs [13]. Thus, active surveillance should be considered on a case-by-case basis, namely in patients presenting with less than 10 mm thickness isthmus PTC.

Microvascular imaging tools such as Super Microvascular Imaging® (SMI) could be more sensitive and specific than conventional US Doppler for the detection of suspicious lymph nodes. This is due to the improvement in detecting very low vascular flows and in the imaging resolution [14].

Interestingly, level I is never involved in case of lymphatic metastatic thyroid spread. US has a low diagnostic sensitivity (around 50% or less) in case of level VI involvement. However particular attention should be paid to lower neck level VI nodes as further lymphatic tumour spread could lead to upper mediastinal lymphadenopathy that will require further thoracic surgery in aggressive tumours. Risk factors for upper mediastinum lymph node metastasis include strong lymphatic involvement of level VI (> 50%) and level IV (> 33%), increased serum Thyroglobulin (Tg) and anti-Tg antibodies level [15].

**Fig. 4:**
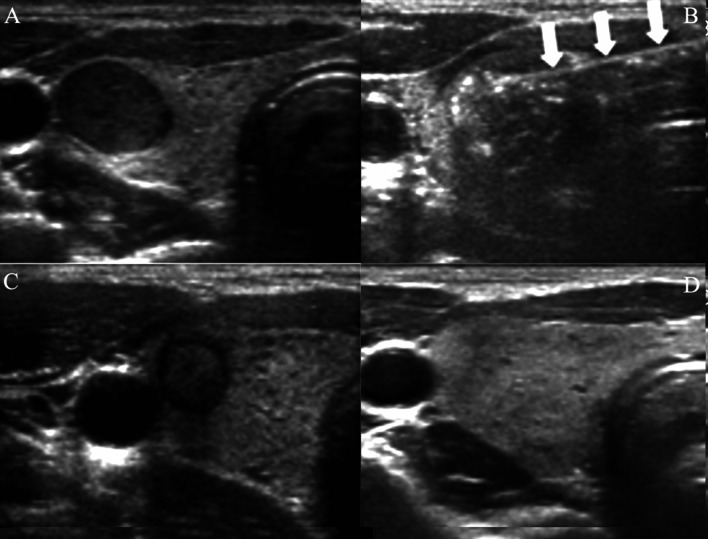
Percutaneous RFA of 11 mm right lobe PTC. **A** Axial US scan of right thyroid PTC. **B** Percutaneous trans isthmic RFA needle (arrows) during RFA treatment. **C** Volume decreases at 3 month follow-up. **D** Complete disappearance at 1 year, without further local recurrence to date. *Courtesy Professor JH Baek (MD, PhD), Republic of South Korea*

Interestingly, percutaneous radiofrequency Ablation (RFA) might represent another safe effective alternative for managing low-risk small PTCs, especially in patients ineligible for surgery. RFA has been advocated as so by the European Thyroid Association and CIRSE guidelines [16] (Fig. 4). A meta-analysis, analysing results of RFA in 1284 PTCs demonstrated that complete disappearance was 76.2% and recurrence rate 0.01% after RFA [17].

**Fig. 5:**
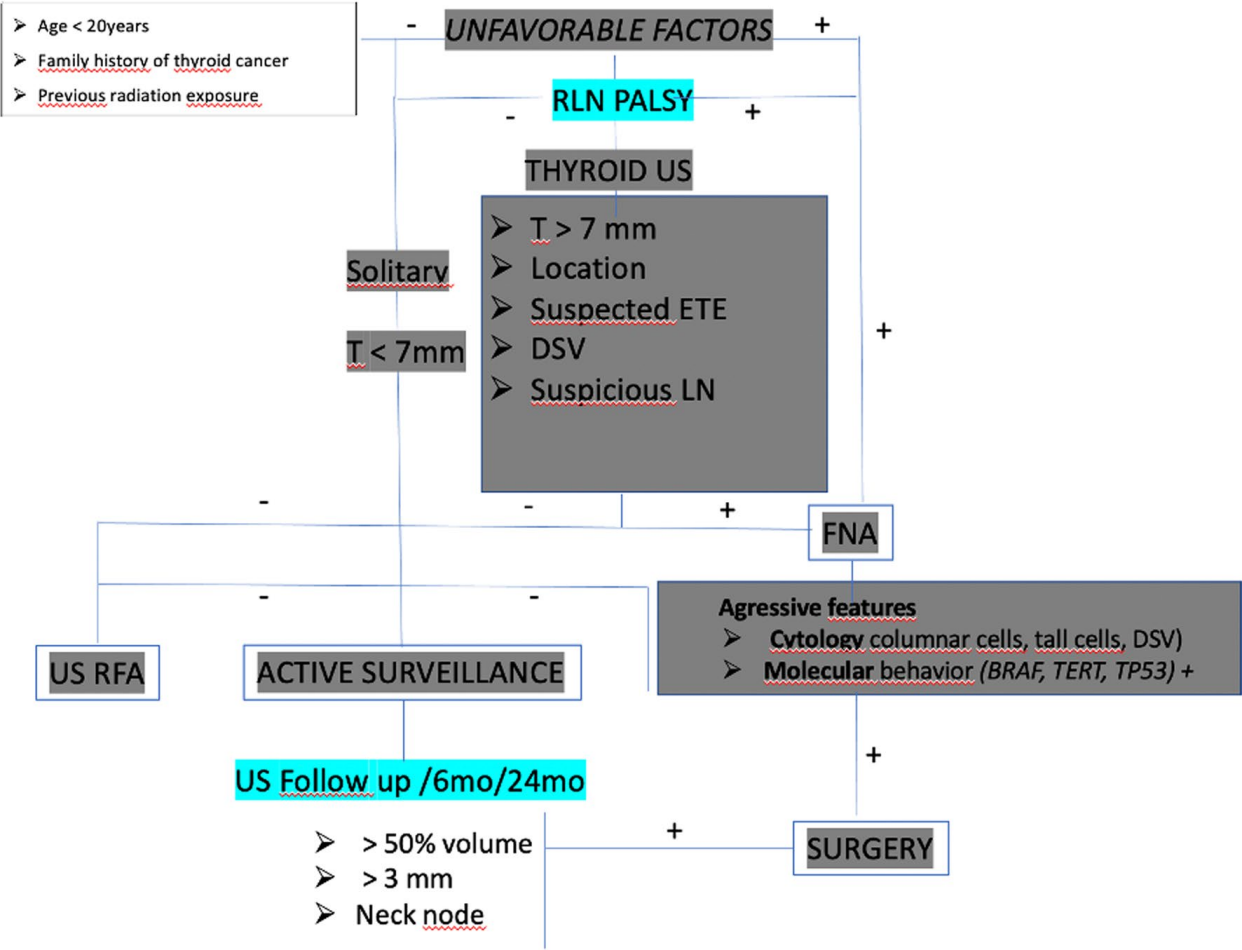
Decision algorithm regarding PTC. Thyroid US is central in decision algorithm of small size PTC. Unfavourable factors, including young age < 20 years, previous radiation exposure and family history of thyroid cancer [18], and recurrent laryngeal nerve (RLN) palsy should lead to thyroid surgery. Five US criteria, including size > 7 mm, particular location, ETE, DSV features, suspicious lymph nodes, associated with aggressive behaviour (cytology, molecular) on FNA should lead to surgery [9, 19]

Thus, a decision algorithm could help the referring physician (Fig. 5). One should consider the existence of a family history [18] of thyroid cancer (5.9%), other unfavourable or aggressive factors leading to surgery, such as cytological/molecular aggressive features [9, 19] the life expectancy and advice of the patient. Finally, even though the vast majority of PTC has an indolent course, some can be revealed by a neck node [20] or exceptionally develop a fulminant lethal course [21]. In all other cases, RFA could be proposed as an alternative to active surveillance and surgery.

## Data Availability

All data and material are included in this Opinion article.

## Abbreviations

ACR TI-RADS: American College of Radiology-Thyroid Imaging Reporting and Data System
ATPO: AntiThyroPerOxidase antibodies
DSV: Diffuse sclerosing variant
ETE: Extrathyroidal extension
Eu-TIRADS: European thyroid imaging reporting and data system
FNA: Fine needle aspiration
MVI: Microvascular imaging
PTC: Papillary thyroid cancer
RFA: Radiofrequency ablation
RLN: Recurrent laryngeal nerve
Tg: Thyroglobulin
US: Ultrasound
